# Neoadjuvant Therapy in Rectal Cancer - Biobanking of Preoperative Tumor Biopsies

**DOI:** 10.1038/srep35589

**Published:** 2016-10-18

**Authors:** Peter Jo, Manuel Nietert, Linda Gusky, Julia Kitz, Lena C. Conradi, Annegret Müller-Dornieden, Philipp Schüler, Hendrik A. Wolff, Josef Rüschoff, Philipp Ströbel, Marian Grade, Torsten Liersch, Tim Beißbarth, Michael B. Ghadimi, Ulrich Sax, Jochen Gaedcke

**Affiliations:** 1 Department of General, Visceral and Pediatric Surgery, University Medical Center, Goettingen, Germany; 2 Department of Medical Statistics, University Medical Center, Goettingen, Germany; 3 Department of Medical Informatics, University Medical Center, Goettingen, Germany; 4 Department of Pathology, University Medical Center, Goettingen, Germany; 5 University Medical Center, Goettingen, Germany; 6 Institute of Pathology, Pathology Nord-Hessen, Kassel, Germany

## Abstract

Translational research relies on high-quality biospecimens. In patients with rectal cancer treated preoperatively with radiochemotherapy tissue based analyses are challenging. To assess quality challenges we analyzed tissue samples taken over the last years in a multicenter setting. We retrospectively evaluated overall 197 patients of the CAO/ARO/AIO-94- and 04-trial with locally advanced rectal cancer that were biopsied preoperatively at the University Medical Center Goettingen as well as in 10 cooperating hospitals in Germany. The cellular content of tumor, mucosa, stroma, necrosis and the amount of isolated DNA and RNA as well as the RNA integrity number (RIN) as quality parameters were evaluated. A high RNA yield (p = 2.75e–07) and the content of tumor (p = 0.004) is significantly associated to high RIN-values, whereas a high content of mucosa (p = 0.07) shows a trend and a high amount of necrosis (p = 0.01) is significantly associated with RNA of poor quality. Correlating biopsies from Goettingen and the cooperating centers showed comparable tumor content results. By taking small sized biopsies we could assess a clear correlation between a good RNA quality and a high amount of RNA and tumor cells. These results also indicate that specimens collected at different centers are of comparable quality.

Large scale tumor sample analyses are generally necessary to identify or validate molecular markers that could help to individualize patient treatment. Beside the tissue that has been retrieved and stored based on rigid standardized operating procedures (SOPs) high quality clinical data are necessary. To provide an excellent platform for translational research tissue acquisition and clinical data collection are best performed within clinical trials^1^. This requires a well-developed information technology (IT) infrastructure^2^ that is of increasing importance if patient numbers are high and combinations of clinical data, biomaterial and molecular results are warranted. However, the introduction of neoadjuvant concepts in the treatment of gastrointestinal tumors such as esophageal^3^, gastric^4^,^5^ and rectal cancer^6^,^7^,^8^,^9^ has led to an increase of demands. In contrast to large tissue samples taken from the surgically resected tumor, biopsies taken prior to the chemotherapy, irradiation or the combination of both pose crucial challenges.

The tiny size of pretherapeutic biopsies dramatically limits the amount of applications requiring multiple biopsies that in turn increase side effects. Drop out rates to biopsy techniques and the interplay between tumor, normal tissue and necrosis has to be considered. Overall, ethical aspects of taking additive tissue that is not required for diagnostic or therapeutic purpose need to be taken into account. Based on two rectal cancer phase III trials (CAO/ARO/AIO-94^6^, -04^10^) the experience from pretherapeutic rectal cancer biopsies taken partially in different hospitals in Germany in a multicenter setting and the potential impact on genome wide screens as well as single protein- and gene- analyses are discussed. Aim of this study was to define data regarding procurement and quality assurance of preoperatively taken biopsies from patients with rectal cancer that can inform the handling of RNAlater biospecimens.

## Material and Methods

### Patients and Multimodality Treatment

Tumor biopsies of overall 197 patients with locally advanced rectal cancer treated with preoperative radiochemotherapy (RCT) collected between 2001 and 2014 at the Department of General, Visceral and Pediatric Surgery at the University Medical Center Goettingen as well as in 10 cooperating hospitals throughout Germany were included for this study. Patients were enrolled in or at least treated according to the CAO/ARO/AIO-94^6^- or CAO/ARO/AIO-04-trial^10^ (EudraCT-Number 2006-002385-20 - NCT00349076) of the German Rectal Cancer Study Group (GRCSG). Written informed consent of all patients participating in the translational study was an inclusion criterion. All experiments were performed in accordance with relevant guidelines and regulations. This study conformed with the ethical principles of the Declaration of Helsinki and was approved by the University of Goettingen Ethics Committee in Goettingen, Germany (application number 20/9/95, 9/8/08). Informed consent was obtained from all patients. The departments are members of the GRCSG and have participated in prospective randomized trials with controlled high quality standard operating procedures for staging, application of RCT surgery and patho-histological work up and received treatment as well as follow-up according to the trial guidelines. Patients were preoperatively treated with RCT followed by surgical resection and pathologic workup standardized according to the guidelines of these randomized phase-III clinical trials. Preoperative RCT included a total radiation dose of 50.4 Gy (single dose of 1.8 Gy) accompanied by either 5-Fluorouracil (5-FU) or a combination of an intravenous infusion of Oxaliplatin and a continuous infusion of 5-FU. Within 4 to 6 weeks after completion of preoperative RCT curative surgery was carried out, including total mesorectal excision. Four to six weeks after surgery treatment was completed with an postoperative therapy with either 5-FU or 5-FU with folinic acid combined with Oxaliplatin. After completion of the multimodal treatment patients were subjected to frequent follow-up examinations. Follow-up data was directly stored accordingly into the clinical database software.

### Ascertainment of Tumor Biopsies and RNA and DNA Isolation

**Figure 1:**
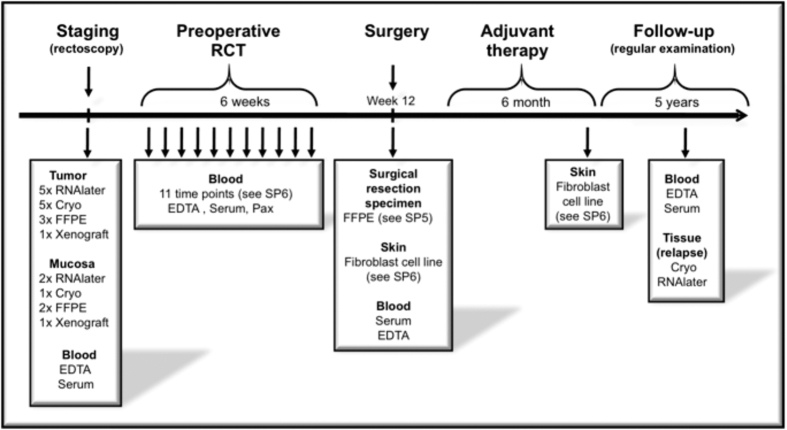
Biomaterial retrieval for a collaborative research initiative.

Pretherapeutic tumor biopsies were collected prospectively by experienced clinicians during the staging procedure. In the beginning of biobanking a single biopsy from the tumor and the surrounding mucosa (at least 3 cm away from the tumor) was taken and stored in RNAlater (Qiagen, Hilden, Germany) according to developed and harmonized SOP’s. RNAlater stabilization reagent stabilizes and protect the RNA expression pattern of samples during harvest and storage. Samples can be archived without risk of RNA degradation, even after multiple freeze–thaw cycles. According to the manufacturer’s recommendations (Qiagen, Hilden, Germany) the samples were kept at room temperature for 24 hours after immersion in RNAlater. This guarantees that the complete sample is absorbed by the reagent. For RNA protection and sample storage, tissue samples were submerged in an appropriate volume of 500 μl of RNAlater reagent immediately (within 5 seconds) after taking the biopsy. After 24 hours in RNAlater reagent at room temperature the samples were kept at −20 °C for archival storage until further processing. With preparing the tumor samples for the first analyses^11^ it became obvious that patients were excluded due to the absence of relevant content of tumor tissue. Accordingly, 3 tumor and 1 mucosa biopsies were taken thenceforward. Starting a collaborative research approach: “Biological Basis of Individual Tumor Response in Patients with Rectal Cancer” funded by the German Research Foundation the “Biomaterial Protocol” was modified again at the Department of General-, Visceral and Pediatric Surgery Goettingen. In this context - in the following years - more tumor, mucosa and liquid biopsies were taken for different research projects and the biomaterial protocol was adapted respectively to the need of each project, lastly modified 2013 in its present form (Fig. 1).

Histo-pathological assessment by board certified pathologists was performed to assess the cellular content (tumor, mucosa, stroma, necrosis) prior to the isolation of nucleic acids. The experienced gastrointestinal pathologists examined in each sample the whole tumor bed and determinded the percentage of tumor by looking at the neoplastic epithelial cells. Biopsies with a tumor content less than 50% were excluded for further molecular analyses. RNA and DNA isolation was performed using TRIZOL (Invitrogen, Carlsbad, CA) as previously described^12^ RNA and DNA amount and quality assessment was conducted using spectrophotometric analysis with the NanoDrop 1000 (Thermo Fisher Scientific, Wilmington, DE, USA). RNA quality assurance was completed by using the RNA integrity number (RIN) as measured by the Agilent BioAnalyzer (Agilent Technologies, Santa Clara, CA). For following molecular analyses samples with a RIN number lower than 5 were excluded. Finally, RNA was stored at −80 °C and DNA at −20 °C, respectively.

### Biobanking Infrastructure and IT Harmonization

Since 2007 the Department of Medical Informatics (MI) has established a professional IT-infrastructure to enable the collection of high-quality biospecimens within the Clinical Research Group 179 (KFO 179) funded by the German Research Foundation. The Good Clinical Practice (GCP)-compliant infrastructure consists of a study database to capture phenotypic data and a biomaterial database for the storage of quality and location information of the biospecimens.

Due to data protection regulations and data handling issues^13^, these collections of clinical and biomaterial data are stored in separate dedicated databases. This minimizes the risk of re-identification of study participants^13^,^14^,^15^. Different identifiers for one study participant are used in the databases. The identifying data will not be stored in the databases. The allocation between the identifying data and the medical data is only possible for authorized personal within the context of treatment. To analyze the data from the different databases it is possible to allocate the study-ID and the biomaterial-ID via a separate list, which is also only accessible for authorized personal with personalized access data. To be in compliance with the GCP guidelines it is necessary for the electronic data capture in clinical trials to have an audit trail, which logs all data changes.

The study data is stored in electronic case report forms (eCRFs) in a GCP and 21 CRF Part11 FDA compliant web based clinical trial data capture system (SecuTrial, iAS, Berlin, Germany). All relevant processes from building and testing to data capture and quality control to the standardized export to biomedical analyses are covered within the research group. Although this infrastructure proved successful during the last 8 years, some further development concerning data integration, data filtering and data analysis^16^ as well as adaptations to the current central UMG biobanking infrastructure^17^,^18^,^19^ have to be addressed in the future.

### Preprocessing and Statistical Analyses

The workflow for the preprocessing and the analysis of the data was implemented using KNIME 2.9.4^20^. KNIME nodes were mostly used for the preprocessing of the data, while we used the R-plugin nodes in KNIME to perform the final statistical tests with R version 3.0.2^21^. The global significance level was set to α = 5%. For comparisons of continuous data we used the Pearson’s correlation coefficient (r). If the data was skewed we used the non-parametric, rank based correlation coefficient (tau) according to Kendall^22^. For comparisons of two continuous data distributions we used the Wilcoxon rank sum test; paired where applicable^23^. In case of three or more different distribution samples we used the Kruskal-Wallis rank sum test^24^ for the comparison. The impact of the ‘% tumor’, ‘RIN’ and ‘quality of sample’ on DFS, CSS and OSS was determined using Kaplan-Meier analysis and assessed for statistical significance using the log rank test and where applicable for the continuous data values using a Cox proportional hazard model^25^. The survival analysis was performed using the R package survival.

## Results

197 patients with locally advanced rectal cancer (UICC II/III) with matched clinical data were evaluated. Of these overall 373 preoperative tumor biopsies were analyzed. The average content of tumor was 60%. In 9% the process of taking the biopsies had to be terminated due to bleeding or patients’ discomfort.

### RNA and DNA yield from RNAlater biopsies

The median RNA and DNA amount that was isolated of a single biopsy was 33.16 μg (minimum 0.01 μg, maximum 5591.2 μg) and 18.885 μg (minimum 0.01 μg, maximum 817.34 μg) respectively (Table 1).

### RIN correlation to isolated RNA yield

**Figure 2:**
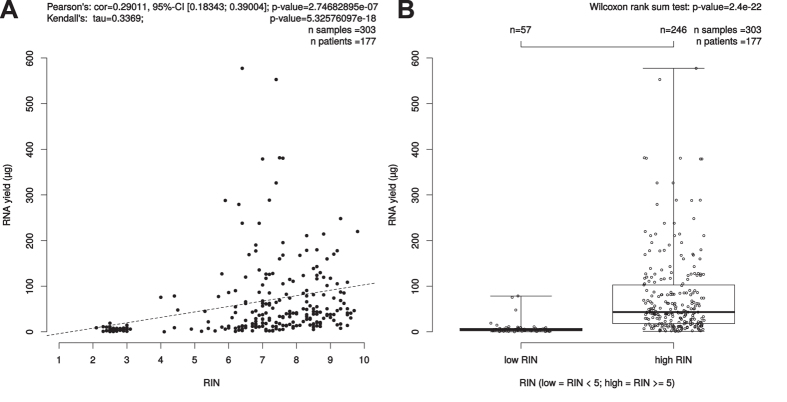
Correlation between isolated RNA yield (μg) and RIN. (**A**) Scatterplot: Pearson’s correlation coefficient (r) and due to skewedness of the data also Kendall’s rank correlation tau were depicted as measures. Kendall’s rank correlation yielded a tau of 0.3369027 and had a p-value of <5.32576097e-18. (**B**) Boxplot: The bimodal distribution of RIN values was the trigger to further investigate the Wilcoxon test results for the low/high RIN classes, which result here in a p-value of 2.4e–22.

To evaluate the role of the RNA quality and the amount of isolated RNA from a single biopsy the RIN-values of 303 RNAlater samples of 177 patients were available for this analysis and were correlated to the isolated RNA yield. A high amount of isolated RNA of one single biopsy was significantly associated to high RIN-values, i.e. good RNA quality (p = 2.747e–07, cor = 0.290, 95%-CI [0.183; 0.390] (Fig. 2).

### RIN correlations to cellular content of tumor, mucosa, stroma and necrosis

**Figure 3:**
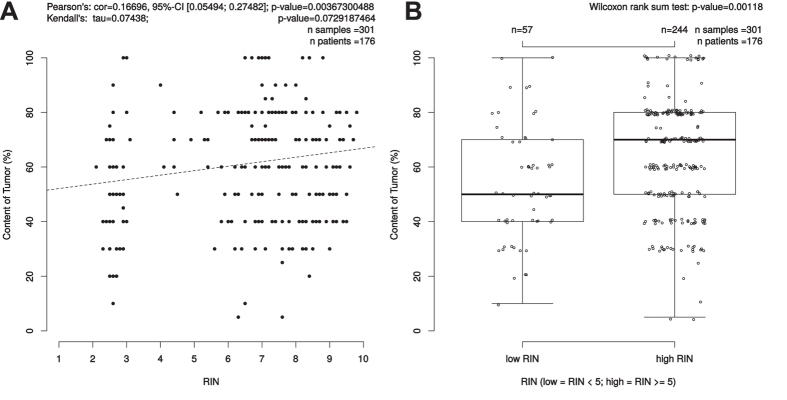
Correlation between content of tumor (%) and RIN. (**A**) Scatterplot: Pearson’s correlation coefficient (r) and due to skewedness of the data also Kendall’s rank correlation tau were depicted as measures. Kendall’s rank correlation yielded a tau of 0.0743838 and had a p-value of 0.07292. (**B**) The bimodal distribution of RIN values was the trigger to further investigate the Wilcoxon test results for the low/high RIN classes, which result here in a p-value of 0.00118.

**Figure 4:**
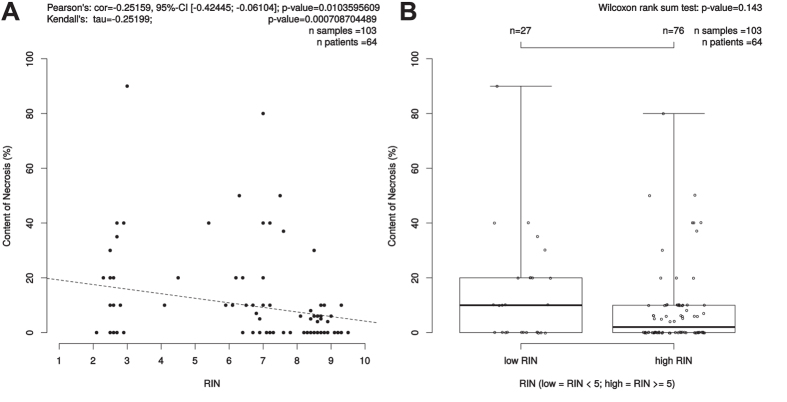
Correlation between content of necrosis (%) and RIN. (**A**) Scatterplot: Pearson’s correlation coefficient (r) and due to skewedness of the data also Kendall’s rank correlation tau were depicted as measures. Kendall’s rank correlation yielded a tau of −0.2519935 and had a p-value of 0.0007087. (**B**) The bimodal distribution of RIN values was the trigger to further investigate the Wilcoxon test results for the low/high RIN classes, which result here in a p-value of 0.143.

To show a potential correlation of RNA quality and the different components of a given tumor we analyzed in all available RNAlater biopsies the correlation between content of tumor, mucosa, stroma and necrosis and RNA quality. Interestingly, the amount of tumor within a biopsy showed a significant, positive correlation with the RNA integrity number (p = 0.004, cor = 0.166, 95%-CI [0.054; 0.275]) (Fig. 3). The content of mucosa showed in 145 samples of 94 patients a trend to RNA with a poor quality (p = 0.07, cor = 0.15, 95%-CI [−0.308; 0.010]) (Supplementary Figure 3). 103 biopsies of 64 patients showed necrosis. Here, low content of necrosis was significantly associated to high RIN-values with p = 0.010, cor = 0.252, 95% CI [−0.424; −0.061] (Fig. 4). There was no association between the content of stroma and RNA quality by analyzing 293 samples of 173 patients (p = 0.3, cor = 0.061, 95%-CI [−0.175; 0.054] (Supplementary Figure 4).

### Relation of tumor content and increased number of biopsies per patient

To identify if the chance for a biopsy with a high tumor cell content increases by an increase of biopsy sampling, patients with more than one biopsy were evaluated. Evaluating only the “best” biopsy of a patient – in fact the one with the highest tumor content – in relation to the number of biopsies taken no correlation was found indicating that the chance to get high tumor content biopsies is not dependent on the number of biopsies taken (p = 0.463) (Supplementary Figure 5). Importantly, there is a clear correlation between the decrease of the median amount of tumor (taken from the multiple biopsies of the same patient) and an increased biopsy number (p < 0.0001) (Supplementary Figure 6).

### RIN correlation between University Medical Center Goettingen and cooperating hospitals

The asservation of rectal cancer biopsies includes biospecimen from the Department of General, Visceral and Pediatric Surgery Goettingen, as well as biospecimen from 10 cooperating hospitals. In comparison to the cooperating hospitals a significant higher number of biopsies were taken in Goettingen. To compare the quality characteristics we analyzed the RIN-values and the tumor content between these centers. Correlating the median (p = 2.59e–07) and maximum (p = 0.0359) RIN-values of the patients from the University Medical Center Goettingen and the 10 cooperating centers showed significant better RNA quality results for the cooperating centers. However, the average RIN value of the University Medical Center Goettingen was still between 5–8 (Supplementary Figures 8 and 9).

Comparing the median and maximum tumor content between the cooperating hospitals and the University Medical Center Goettingen showed no significant difference with p-values of p = 0.197 and p = 0.635 respectively (Supplementary Figures 10 and 11).

### Excluded biospecimen and prognosis

**Figure 5:**
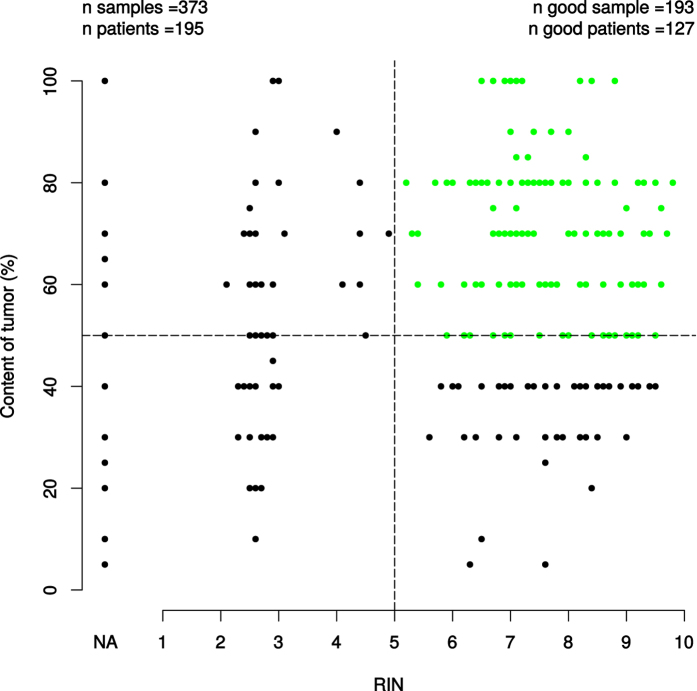
Of 195 analyzed patients (matched to available clinical data sets) 127 patients (65.13%) (good patients) were included for further molecular analyses (tumor content > 50%, RIN > 5). RNAlater biopsy samples not fullfilling the criterions (tumor content < 50%, RIN < 5) were excluded (patients n = 68, 34.87%). The RIN = NA depicts missing RIN-values in the set, which led to exclusion in subsequent RIN vs X analysis.

Of 195 analyzed patients matched to available clinical data sets 127 patients (65.13%) were included for further molecular analyses (tumor content > 50%, RIN > 5). RNAlater biopsy- or RNA samples not fulfilling the criterions (tumor content < 50%, RIN < 5) were excluded (patients n = 68, 34.87%) (Fig. 5).

**Figure 6:**
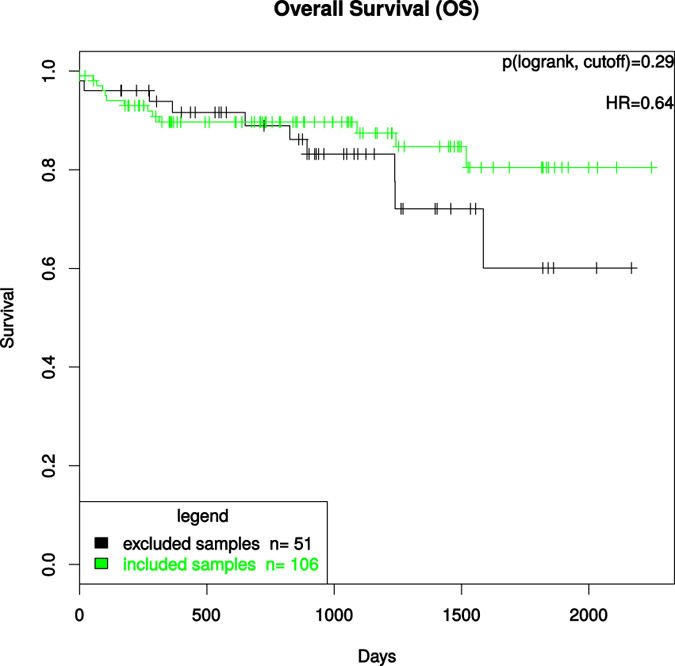
Kaplan-Meier-Curve displaying the OS between the excluded and included patients for further molecular analyses. The test used was the log rank test for differences regarding survival in “excluded samples” vs “included samples” groups, which were defined by cutoffs (tumor content > 50% and RIN > 5).

**Figure 7:**
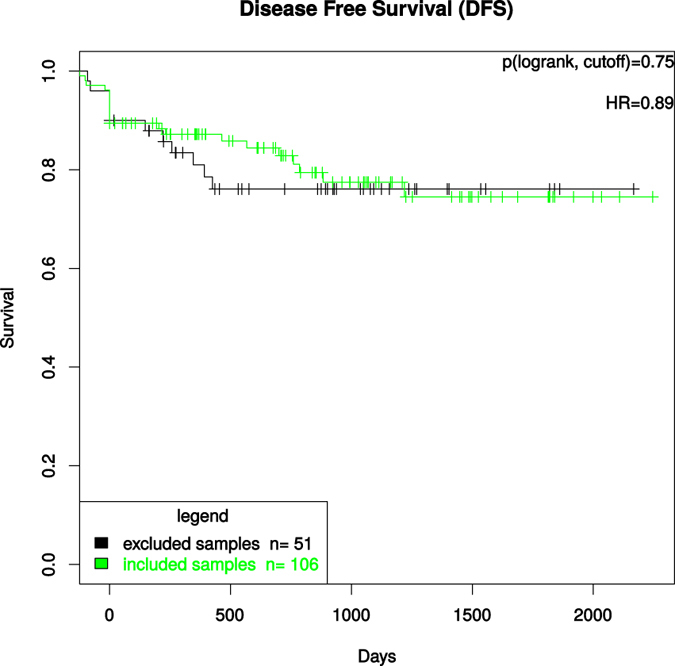
Kaplan-Meier-Curve displaying the DFS between the excluded and included patients for further molecular analyses. The test used was the log rank test for differences regarding survival in the “excluded samples” vs “included samples” groups, which were defined by cutoffs (tumor content > 50% and RIN > 5).

To exclude a potential correlation between prognosis and inadequate biospecimen quality we correlated the patients’ biopsies who were excluded for molecular analyses due to low tumor content (tumor content < 50%) and/or low RIN-values (RIN < 5) with survival parameters. Here we could not find a significant correlation between the survival parameters overall- (OS) and disease-free-survival (DFS) (Figs 6 and 7).

## Discussion

The majority of samples in existing biorepositories are surgical specimens of primary tumors or metastases. Due to the shift towards neoadjuvant treatment protocols biobanking of tissue taken prior to surgical procedures has become a challenging task. As a prerequisite for storage, all biospies were worked-up histopathologically. Interestingly, about one third of all samples macroscopically considered as tumor biopsies did not contain tumor tissue. This unexpected high dropout rate might be due to the procedure of rigid rectoscopy. The rectoscope itself typically consists of a single hollow channel with a removable optic-lens at the end towards the endoscopeur. To take the biopsy the optic-lens is removed and the biopsy forceps is inserted through the hollow channel. The macroscopic visualization of the tumor is clearly reduced by this and might cause false tumor biopsy sampling even in experienced hands. Within the remaining two thirds of the biopsies, however, it has to be noticed that the amount of tumor content ranged between >0% and 100% next to the tumor tissue, normal rectal epithelium, necrosis and stroma. This mixture of tissue shows the effects of the biopsy itself but also the architecture of rectal cancer. The absence of high tumor content in every single biopsy has previously been reported by Webster *et al*.^26^. They compared lymphoma, melanoma and osteosarcoma and demonstrated a highly variable tissue composition. While lymphoma showed a high degree of tumor in each tissue sample the median tumor content for osteosarcoma ranged in an interquartile range of 44% and a medium area occupied by tumor of 39%. On the basis of small amounts of tissue and the low amount of retrieved nucleic acids that ranged from 0.1 μg to 5591.2 μg for RNA (median: 33 μg) and from 0.01 to 817.34 μg for DNA (median: 19 μg) that is derived from 197 patients it is evident that a higher number of biopsy samples need to be taken. Combining this finding with the need of high tumor percentage in the biopsies a first approach would be to increase the number of biopsies. Nevertheless, we were able to show that this does not automatically increase the probability of good tumor content biopsies. And in contrast to what could be assumed, the increased number of biopsies reduced the median tumor content. This may be at least in part explained by the effects of multiple biopsies such as bleeding reducing the general view on the tumor and in consequence impairing the conditions for the biopsy. Therefore, microdissection could be another option. However, for RNAlater probes this is a problematic procedure and was not successful in our hands.

Tumor content is not the only relevant item when working with patient material. The quality such as the RNA integrity, referred to as RIN, is of relevance. Even though the standardized procedure of taking tumor samples adds only small variances that influence the RIN, we interestingly identified the accompanying tissue in the biopsy as a significantly correlated factor. Increased content of mucosa or necrosis decreased the RIN. As a potential explanation we hypothesize that in contrast to tumor it adds contamination by stool and cell detritus that was not irrigated during the preparation of the rectum before rectoscopy. The correlation between the amount of retrieved RNA and its quality is of interest as if this is due to the size of the biopsy this could be influenced by the choice of forceps to perform the biopsy. RNA is a thermodynamically stable molecule, which is, however, rapidly digested in the presence of the nearly ubiquitous RNase enzymes. As a result, shorter fragments of RNA commonly occur in a sample, which can potentially compromise results of downstream applications. One explanation for the association of low RNA yield with lower RIN-values might be the susceptibility of smaller samples (and thus lower RNA yield) to RNase enzymes, specially during the RNA isolation process. To be noted the degradation process of RNA is still only partly understood since it is dependent on the type of RNase that is present and is often combined with fragmentation processes. Alternatively, this could only reflect the tumor tissue content assuming that tumor cells have more RNA than mucosa or stromal cells. Independently of the reasons for this correlation that will in the future be analyzed by weighing and measuring the size of the biopsy the application of larger forceps is critical with respect to bleeding if multiple biopsies are retrieved. To avoid a systematical effect such as low tumor cellularity or poor RNA quality in association with poorer outcome by excluding these tumor samples we correlated these samples to survival parameters. However, no correlation was found indicating that these samples can be taken out without including technical bias in molecular analyzes. Thus the exclusion of patients due to sample inadequacy does not create a selection bias for those patients that would be used for further translational analyses.

Independently of the number, it should be considered that each biopsy prior to the therapy is taken additionally. As diagnosis and therapy do not depend on these tissues, discomforts due to the biopsy itself as well as potential side effects need to be clearly illustrated with written informed consent for patients^27^. This is in clear contrast to biomaterial retrieved from surgical specimens that are resected anyway. With respect to side effects, fortunately, bleeding may become the only severe problem by taking biopsies from patients with rectal cancer. Anatomically, rectal cancers of the lower and middle part of the rectum are surrounded by fatty tissue, called the mesorectum. Due to this anatomic characteristic a potential perforation of the rectum does not lead to a free perforation of a hollow organ. This is in contrast to rectal cancer of the upper rectum, the esophagus or the stomach. Although theoretically possible for tumors in between the upper and the middle third of the rectum this did not occur in a single patient. In contrast, bleeding is a realistic and relevant complication if it is severe. Aggravated by systemic anticoagulation that is continuously given during staging procedures the probability of bleeding increases by multiple biopsy taking. In our experience multiple biopsy taking was stopped in 9% due to small bleedings without any further complication with one exception of a bleeding requiring endoscopical intervention. In this particular case a suprarenin saturated tamponade was inserted and the bleeding successfully stopped. Although taking multiple biopsies can be considered as safe procedures, it implies that it should be performed with caution in outpatient clinics. As general consideration the sequence of fixation media into which the biopsies are transferred deserves a closer attention. If the process of biopsy taking unexpectedly needs to be stopped the most important biopsies should already be transferred. Thereafter the distribution of biopsies on the specific fixation media should be standardized.

In summary, taking multiple pretherapeutic biopsies from rectal cancer is a safe procedure. The technique itself has limitations as it is not performed under direct visual control. The varying amount of tumor in each biopsy is associated to RNA quality and needs to be assessed prior to the analyses. Depending on the intended analyses fixation media and thresholds of tumor cell content and quality parameters impact the number of biopsies needed. We present for the first time data regarding procurement and quality assurance of preoperatively taken biopsies from patients with rectal cancer that can inform the handling of RNAlater biospecimens in a multicenter setting. By taking small sized biopsies preoperatively we could assess a clear correlation between a good RNA quality and a high amount of RNA and tumor cells as well. When taking tumor biopsies contamination with mucosa or necrotic tissue should be avoided since the RNA quality is influenced negatively. These results also indicate that specimens collected at different centers are of comparable quality and can serve as an adequate resource to create a national cohort for the investigation of molecular biomarkers in rectal cancer. Tumor biopsies taken by an experienced clinician with inadequate tumor content and/or RIN-values should be excluded from further molecular analyses.

## Additional Information

**How to cite this article**: Jo, P. *et al*. Neoadjuvant Therapy in Rectal Cancer - Biobanking of Preoperative Tumor Biopsies. *Sci. Rep.* **6**, 35589; doi: 10.1038/srep35589 (2016).

## Supplementary Material

Supplementary Information

## Figures and Tables

**Table 1:** Trizol isolated RNA and DNA amount from RNAlater biopsies.

	RNA Amount	DNA Amount
Minimum	0.1	0.01
Smallest	0.1	0.01
Lower Quartile	10.08	3.93
Median	33.16	18.885
Upper Quartile	79.43	45.74
Largest	183.24	108.08
Maximum	5591.2	817.34
