# Supplementary material for: Neoadjuvant Therapy in Rectal Cancer - Biobanking of Preoperative Tumor Biopsies
Source: Sci Rep. 2016 Oct 18;6:35589. doi: 10.1038/srep35589 (PMC5067705; doi:10.1038/srep35589)
Supplement: Supplementary Information [file srep35589-s1.pdf]

Supplementary Information

Neoadjuvant Therapy in Rectal Cancer

# - Biobanking of Preoperative Tumor Biopsies

Peter Jo MD[1], Manuel Nietert PhD[2], Linda Gusky[3], Julia Kitz MD[4], Lena C. Conradi MD[1], Annegret Müller-Dornieden MD[1], Philipp Schüler MD[1], Hendrik A. Wolff MD[5], Josef Rüschoff MD[6], Philipp Ströbel MD[4], Marian Grade MD[1], Torsten Liersch MD[1], Tim Beißbarth PhD[2], Michael B. Ghadimi MD[1], Ulrich Sax PhD[3], Jochen Gaedcke MD[1#]

[1]Department of General, Visceral and Pediatric Surgery, University Medical Center, Goettingen, Germany

[2]Department of Medical Statistics, University Medical Center, Goettingen, Germany

[3]Department of Medical Informatics, University Medical Center, Goettingen, Germany

[4]Department of Pathology, University Medical Center, Goettingen, Germany

[5]University Medical Center, Goettingen, Germany

[6]Institute of Pathology, Pathology Nord-Hessen, Kassel, Germany

[#] corresponding author

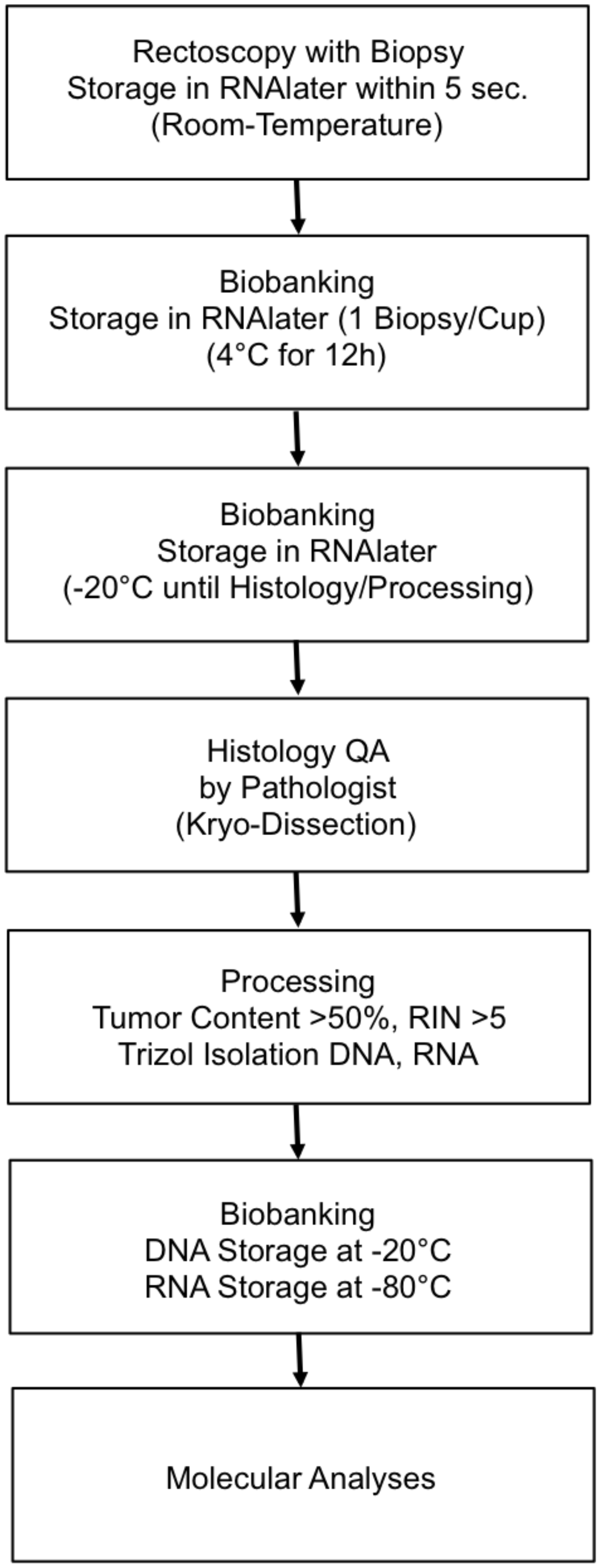

Supplementary Figure 1: RNAlater biopsy processing workflow

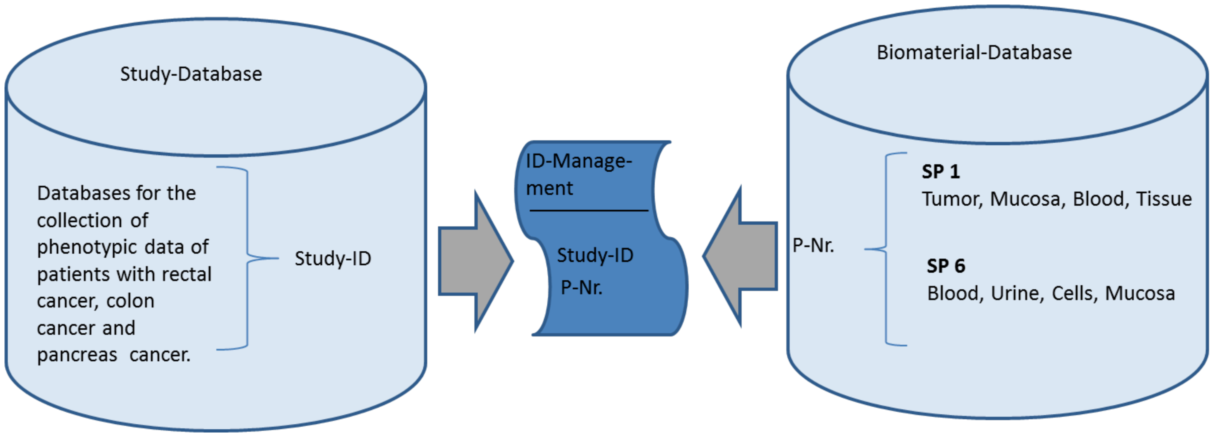

Supplementary Figure 2: Simplified sketch of the IT-Infrastructure of the KFO 179: separated storage for trial data and biomaterial data combined via a simple pseudonymization protocol following the TMF guidelines (Technologie- und Methodenplattform für die vernetzte medizinische Forschung - umbrella organization for networked medical research in Germany; (ID – Identification, P-Nr. – Patientnumber, SP – Subproject within the KFO 179

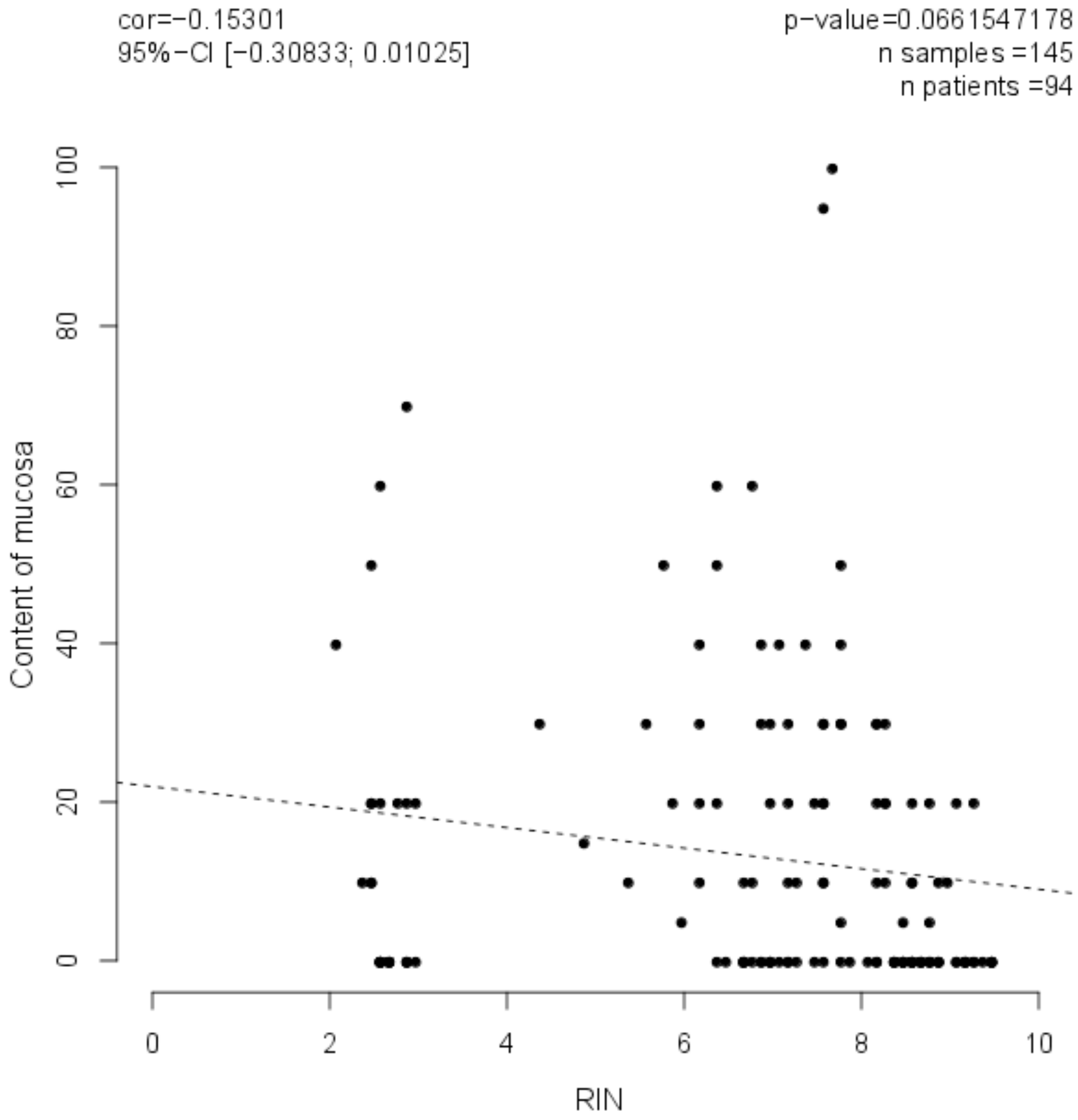

Supplementary Figure 3: Correlation between content of mucosa (%) and RIN

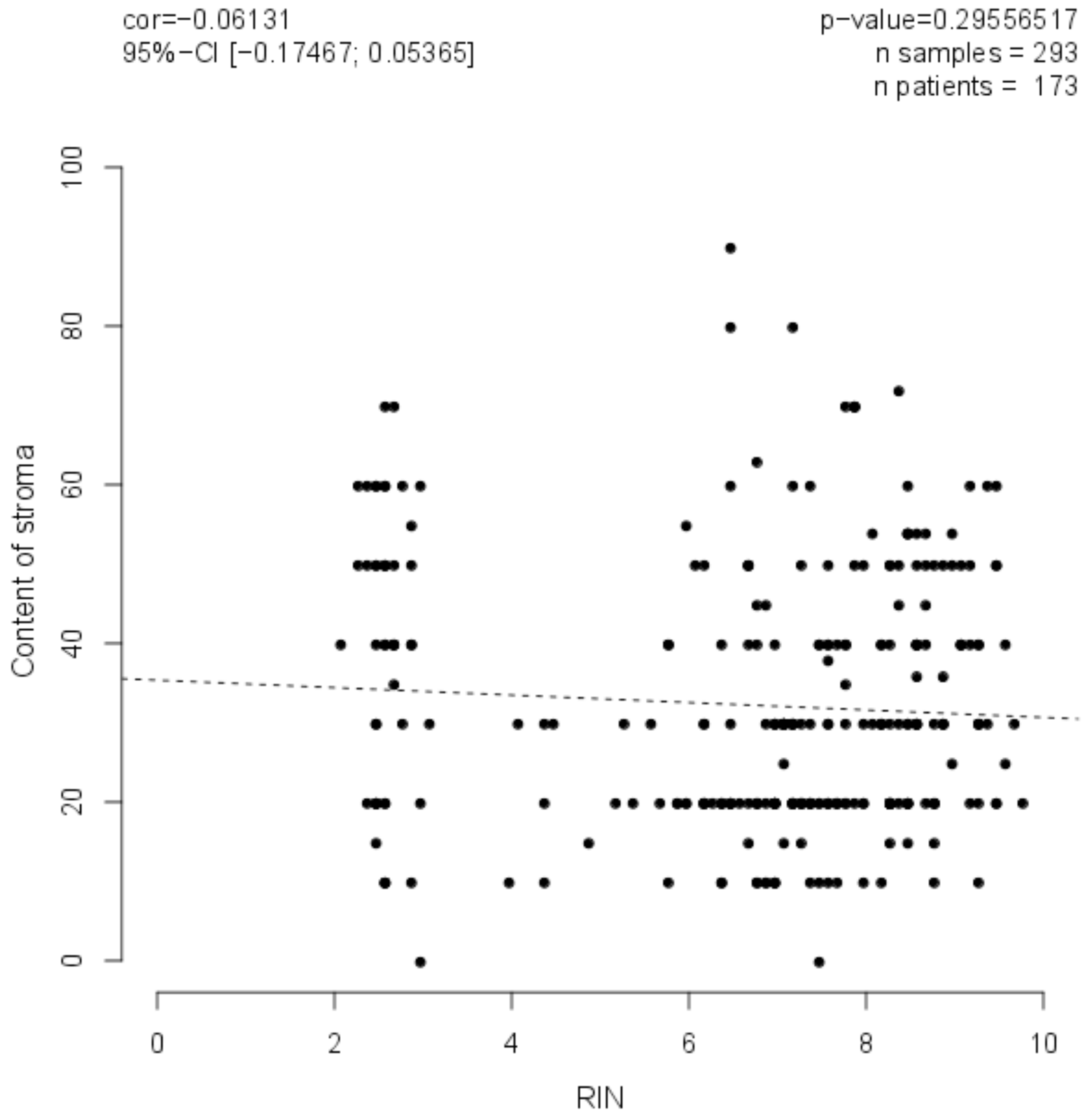

Supplementary Figure 4: Correlation between content of stroma (%) and RIN

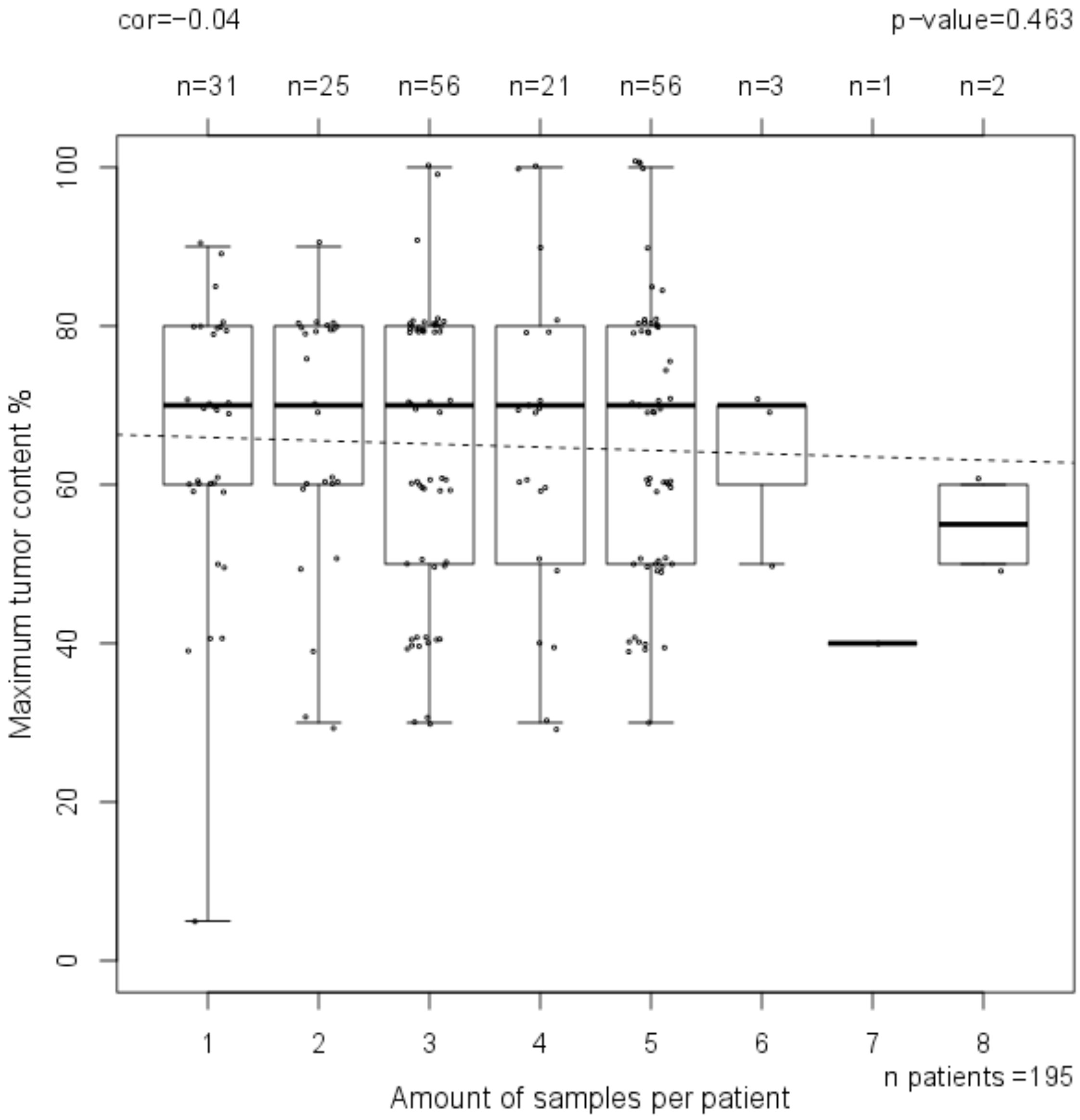

Supplementary Figure 5: Correlation between amount of probes per patient versus maximal tumor content (%) (only the biopsy with the highest tumor content of a patient was plotted)

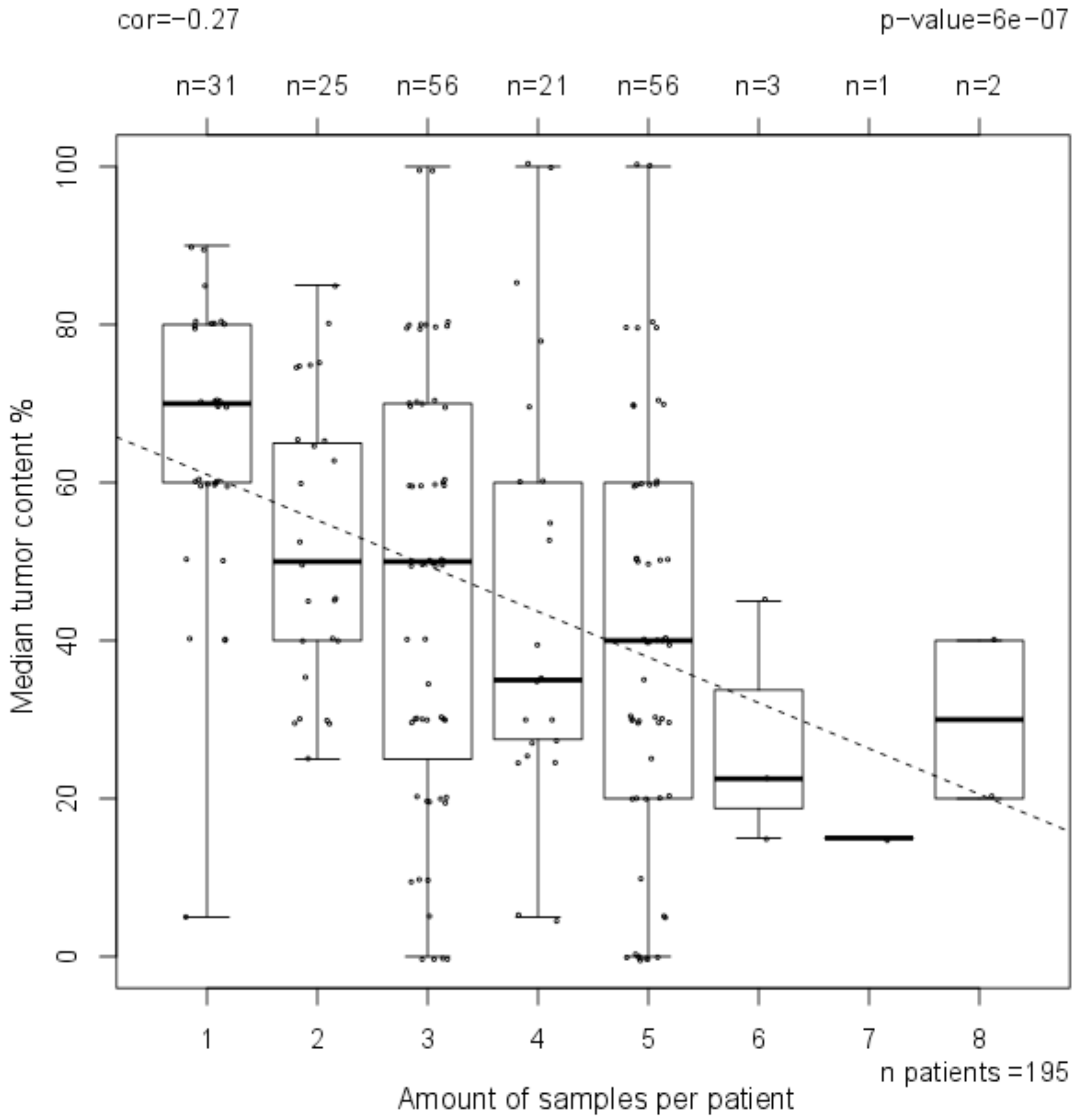

Supplementary Figure 6: Correlation between amount of probes per patient versus median tumor content (%)

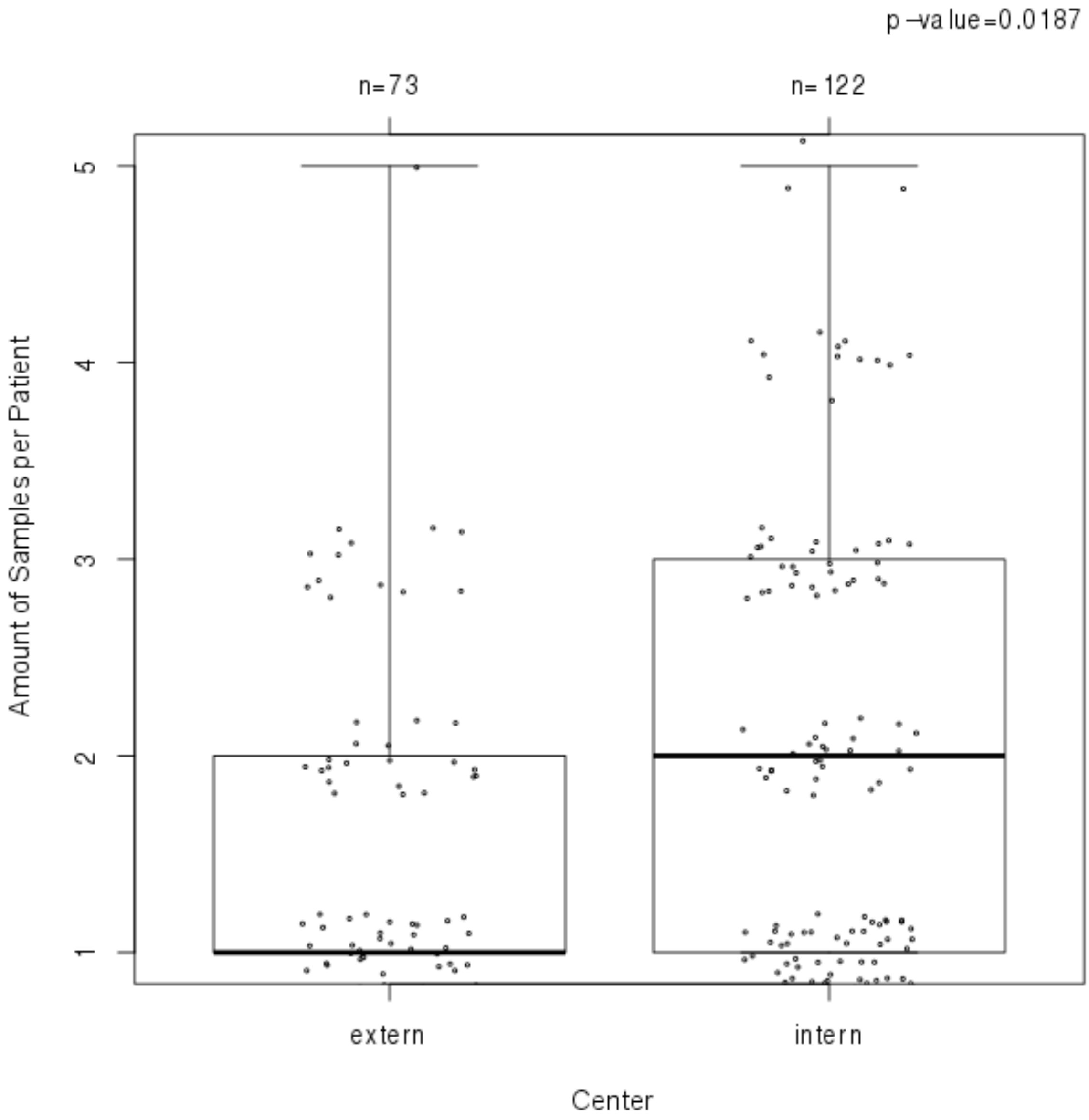

Supplementary Figure 7: Distribution of the amount of Samples between the cooperating hospitals (extern) and the Department of General-, Visceral and Pediatric Surgery Goettingen (intern)

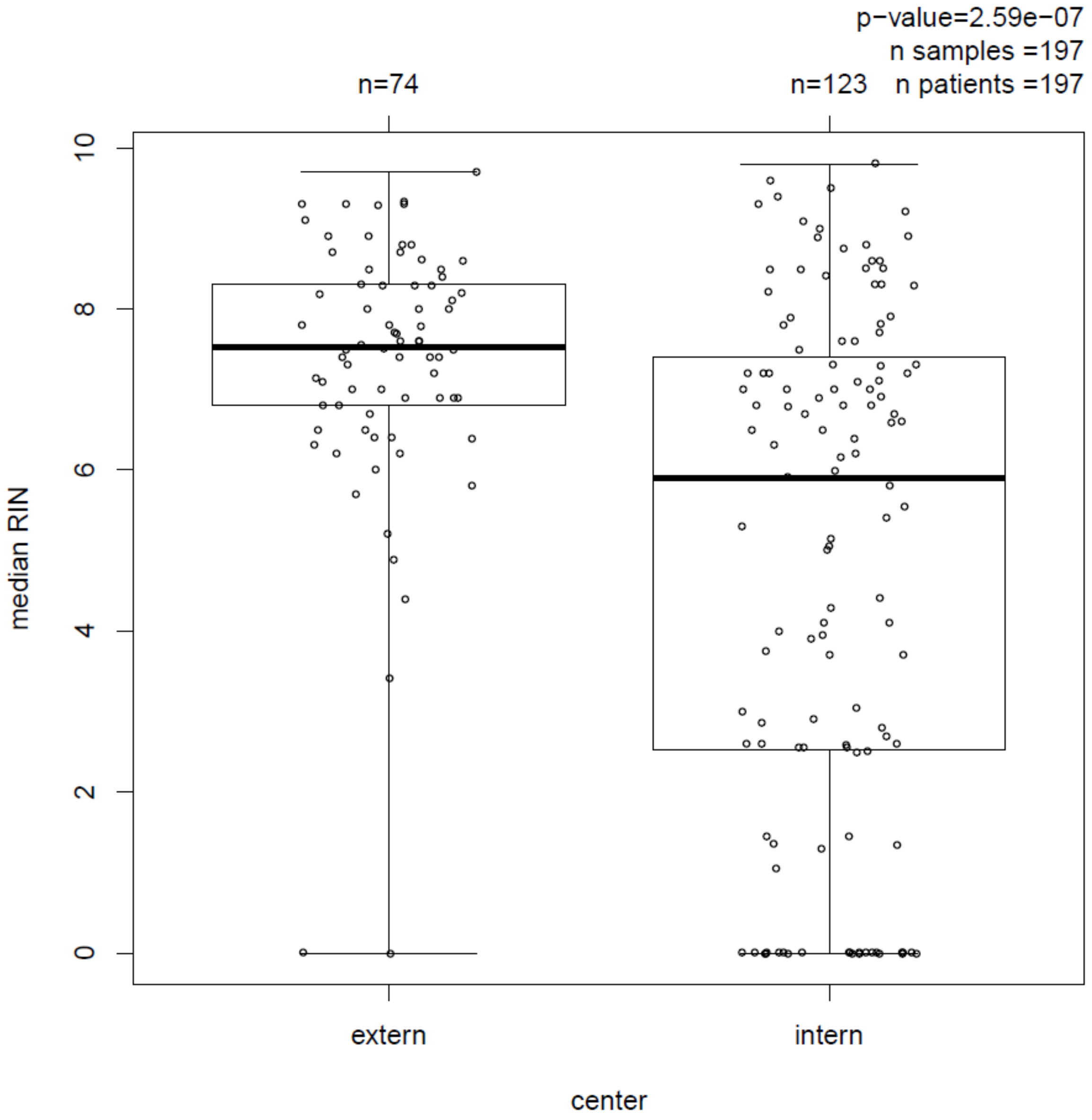

Supplementary Figure 8: Correlation between the median RIN, the different centers (extern) and the University Medical Center Goettingen (intern)

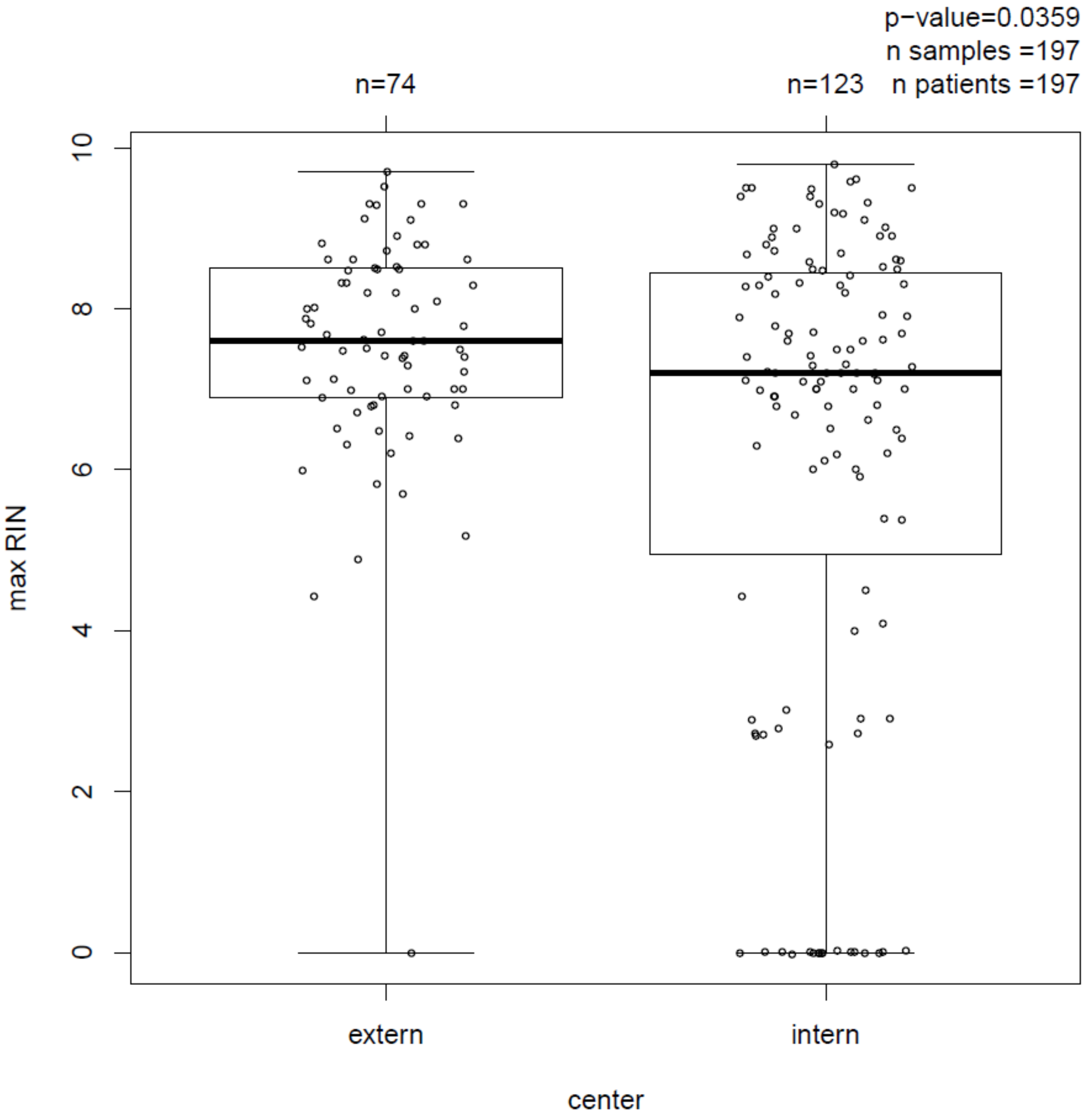

Supplementary Figure 9: Correlation between the maximum (max) RIN, the different centers (extern) and the University Medical Center Goettingen (intern)

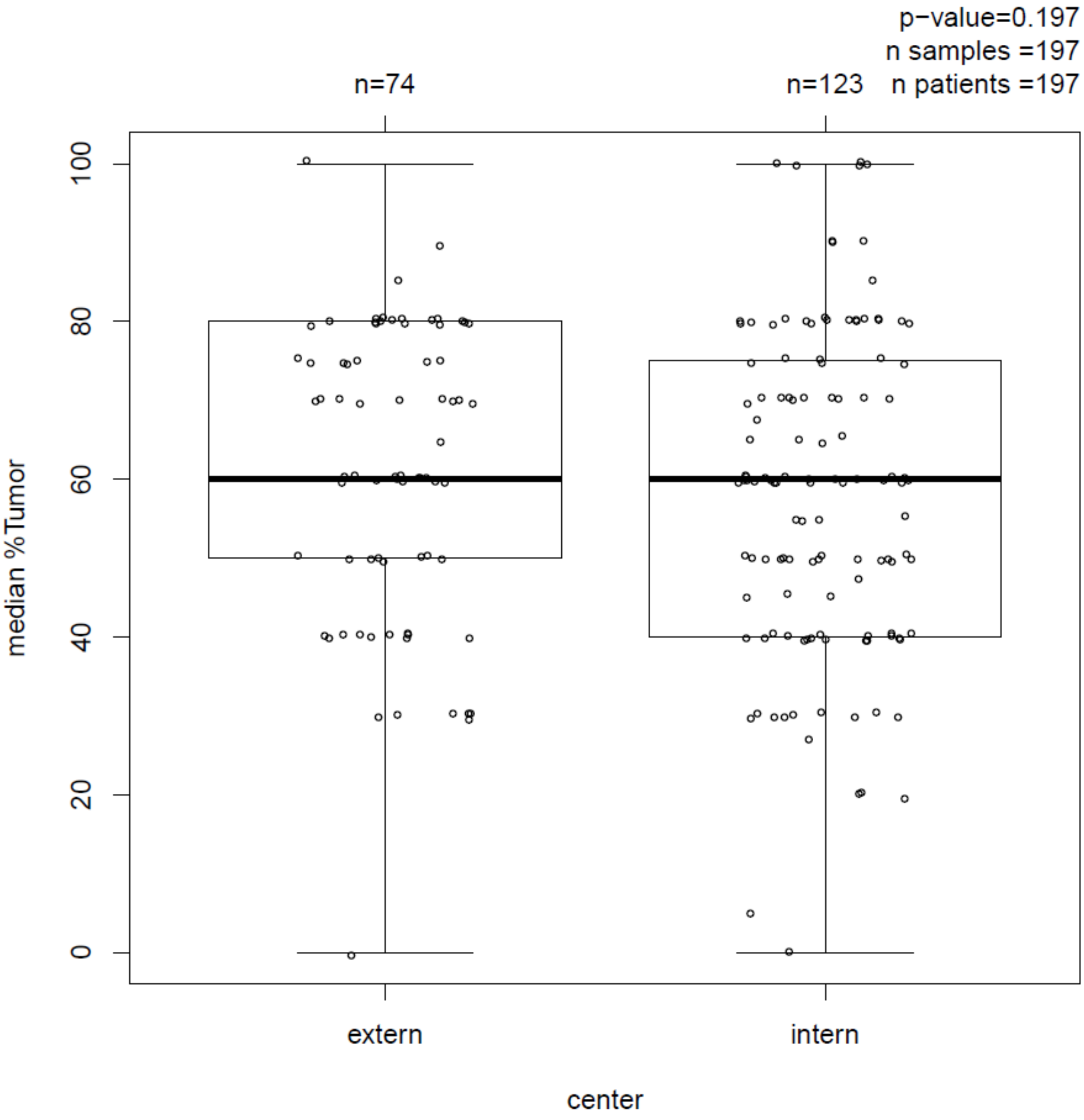

Supplementary Figure 10: Correlation between the median tumor content (%), the different centers (extern) and the University Medical Center Goettingen (intern)

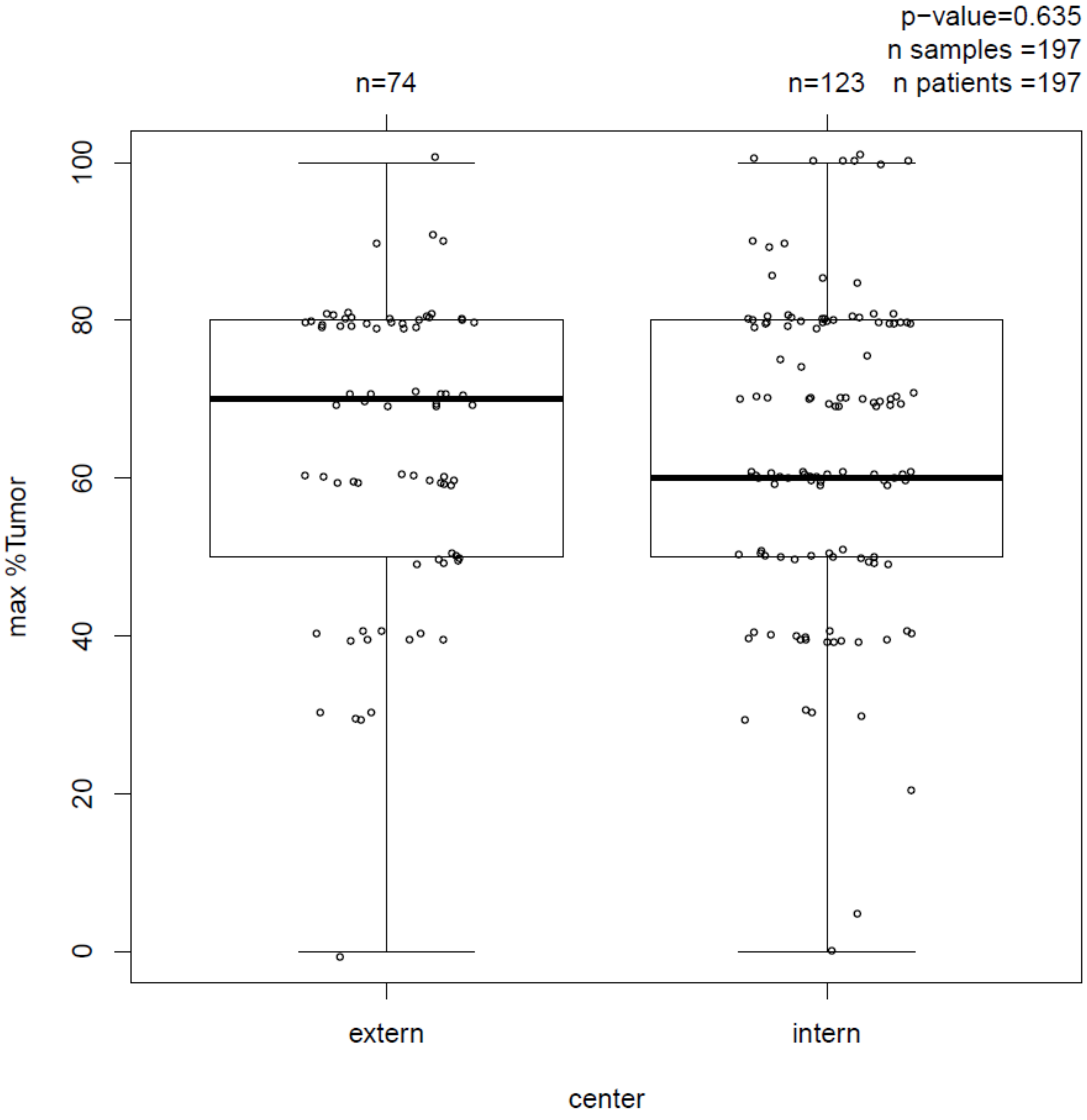

Supplementary Figure 11: Correlation between the maximum (max) tumor content (%), the different centers (extern) and the University Medical Center Goettingen (intern)
